# Catheter Ablation of Left Ventricular Summit Arrhythmia in a Patient with Critical Coronary Artery Stenosis: A Sequential Approach

**DOI:** 10.19102/icrm.2020.111004

**Published:** 2020-10-15

**Authors:** Gökhan Aksan

**Affiliations:** ^1^Department of Cardiology, Samsun Education and Research Hospital, Samsun, Turkey

**Keywords:** Left ventricular summit, premature ventricular contraction, radiofrequency catheter ablation

## Abstract

The left ventricular (LV) summit is the usual source of epicardial idiopathic premature ventricular contractions (PVCs). A 56-year-old male patient presented to the cardiology outpatient clinic with palpitations and dyspnea. Twelve-lead electrocardiography performed on admission revealed monomorphic PVCs with precordial QRS transition in the V1 derivation and an rS pattern in the D1 derivation and inferior axis. An electrophysiology study and ablation procedure were planned. Activation mapping guided by a three-dimensional electroanatomic system was conducted to identify the earliest site of ventricular activation of the PVCs. During the PVCs, the earliest ventricular activation was observed within the great cardiac vein (GCV) and preceded the QRS onset by 37 ms. Coronary angiography was performed before ablation in the coronary venous system (CVS) to assess the distance from the coronary artery, which showed severe stenosis in the left circumflex artery. Then, percutaneous coronary intervention was performed to address the left circumflex artery stenosis. Anatomic catheter ablation was performed in the aortic cusp and endocardial LV outflow tract, the sites adjacent to the LV-summit PVC origin. However, successful ablation could not be achieved. Subsequently, an irrigated radiofrequency current was delivered in the GCV for 60 seconds, with the power being gradually increased to 30 W and with an irrigation flow rate of 30 mL/min. After ablation, under isoproterenol infusion and burst pacing from the right ventricle, no PVC or ventricular tachycardia was observed. Special precautions should be taken to avoid coronary artery damage during ablation from distal CVS. This approach may increase the success of ablation and avoid potential complications.

## Introduction

Radiofrequency (RF) catheter ablation is commonly used to treat idiopathic ventricular arrhythmia (VA). Although RF catheter ablation is an effective and successful method for the treatment of outflow-tract VA, it has a low likelihood of success in treating epicardial and mid-myocardial VA.^1,2^ The left ventricular (LV) summit is the common origin of epicardial idiopathic VAs.^3^ However, close proximity to the coronary arteries and anatomic barriers such as epicardial adipose tissue may pose challenges in the mapping and catheter ablation of VAs with an LV summit origin.^2,4^ The earliest activation sites of VAs originating from the epicardial side of the outflow tracts may be recorded within the coronary venous system (CVS). Ablation of these VAs may be successfully performed with RF catheter ablation from within the CVS.^5^ In some cases, catheter ablation through the CVS may not be possible due to difficulties in advancing the catheter within the CVS, close proximity to epicardial coronary arteries, and poor energy delivery.^6^ In such cases, catheter ablation from the endocardial region closest to the LV summit VA origin (anatomic approach) has been reported to produce successful outcomes.^7,8^

This case report presents a stepwise treatment approach to managing an epicardial premature ventricular contraction (PVC) of the LV summit origin with adjacent critical coronary artery stenosis.

## Case report

**Figure 1:**
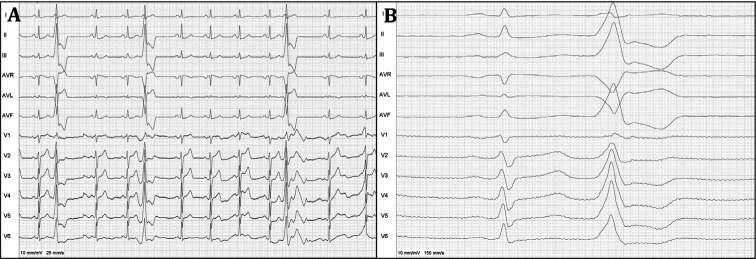
**A:** Twelve-lead ECG at 10 mm/mV 25 mm/s of the patient on admission. **B:** Twelve-lead ECG at 10 mm/mV 150 mm/s of the patients on EPS. ECG: electrocardiogram; EPS: electrophysiology study.

A 56-year-old male patient presented to the cardiology outpatient clinic with palpitation, dyspnea, and chest pain. A 12-lead electrocardiography (ECG) on admission revealed monomorphic PVCs with precordial QRS transition in the V1 derivation and rS pattern in the D1 derivation and inferior axis **(Figures 1A and 1B)**. Twenty-four-hour ambulatory ECG monitoring demonstrated frequent monomorphic bigeminal and trigeminal PVCs (26.045 beats, with 23.7% PVC burden). No episode of ventricular tachycardia (VT) was observed. Class I antiarrhythmic agents (150 mg of propafenone hydrochloride twice daily), taken for approximately three years, were ineffective on these symptomatic PVCs. Transthoracic echocardiography revealed that the LV was dilated with an ejection fraction of 48%, per the modified Simpson method, and showed mild global hypokinesia. Because of the patient’s cardiovascular risk factors (smoking and hypertension), an exercise stress test was performed for ischemic etiology before PVC ablation. In the maximal stage, the patient did not have chest pain or ischemic ST-segment deviation. Meanwhile, the Duke treadmill score was +11 (low risk). Furthermore, during the exercise stress test, the PVC morphology did not transform from monomorphic to polymorphic.

**Figure 2:**
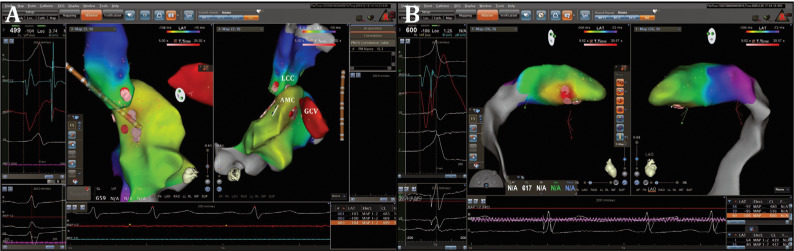
**A:** Simultaneous anatomic and activation mapping performed in the LCC, endocardial LVOT, and GCV using a 3D electroanatomic mapping system during PVC. **B:** Simultaneous anatomic and activation mapping within the CVS. During the PVC, the earliest ventricular activation was observed within the CVS and preceded the QRS onset by 37 ms.

**Figure 3:**
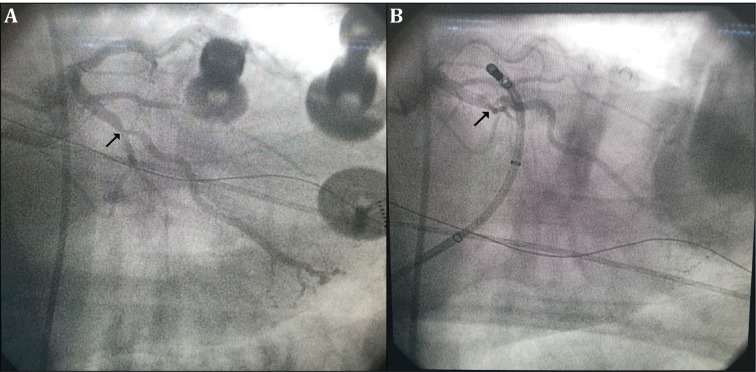
**A:** Fluoroscopic image exhibiting critical coronary artery stenosis in the proximal LCX (black arrow). **B:** Fluoroscopic image of the ablation catheter in the CVS adjacent to the critical coronary artery stenosis (black arrow).

After obtaining informed consent, an electrophysiology study (EPS) was performed with the patient in a fasting, nonsedated state. Antiarrhythmic agents were discontinued for at least five half-lives before the study. Baseline ECG during the study demonstrated frequent PVCs identical to the clinical PVC morphology. Via the right femoral vein, a quadripolar catheter and a deflectable decapolar catheter were placed in the right ventricular (RV) apex and the coronary sinus (CS), respectively. Both anatomic and activation mapping were performed using a three-dimensional (3D) electroanatomic mapping system with a 3.5-mm open-irrigated-tip catheter (ThermoCool; Biosense Webster, Diamond Bar, CA, USA). During LV mapping, heparin was intravenously administered to maintain an activated clotting time of greater than 300 ms. The CS catheter was advanced further into the great cardiac vein (GCV) as much as possible until the proximal electrode pair recorded a ventricular activation earlier than of the distal electrode pair during the clinical PVC.^9^ The activation time on the mapping catheter was measured from the onset of the bipolar electrogram on the distal bipole to the earliest onset of the QRS complex in any of the ECG leads. The unipolar electrograms were also analyzed to determine which bipole pairs were the earliest. During PVC, activation mapping was performed in the aortic cusps, endocardial LV outflow tract (LVOT), and distal CS **(Figure 2A)**, while the earliest ventricular activation observed within the GCV that preceded the QRS onset by 37 ms was recorded on the bipolar recording with a steep QS pattern on the unipolar recording **(Figure 2B)**. Before ablation in the CVS, coronary angiography (CAG) was performed with the femoral approach using the Judkins technique to determine the clearance of the ablation target site according to the proximity of the coronary arteries. CAG showed severe stenosis in the proximal left circumflex artery (LCX) **(Figures 3A and 3B)**.

**Figure 4:**
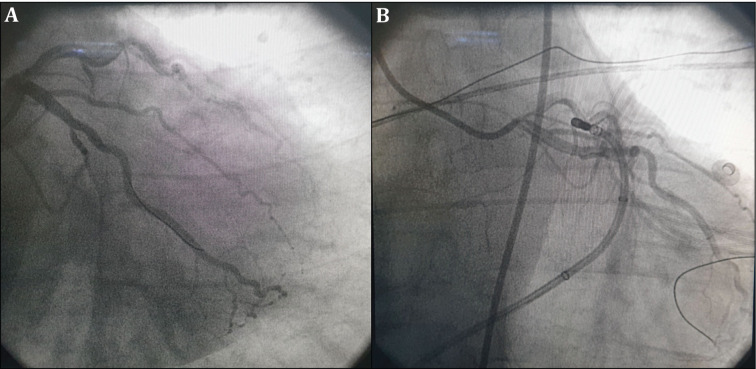
**A:** Fluoroscopic image after PCI of the critical coronary artery stenosis in the proximal LCX. **B:** Fluoroscopic image of the ablation catheter at the successful ablation site.

**Figure 5:**
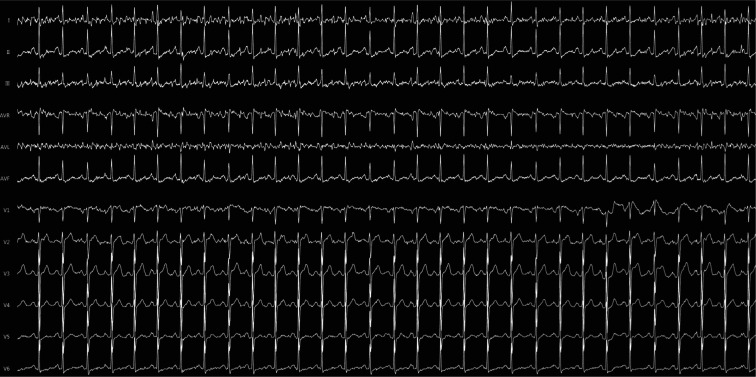
Postablation 12-lead surface ECG.

First, percutaneous coronary intervention (PCI) was planned for the LCX coronary artery stenosis. The proximal LCX coronary artery stenosis was predilated with a 2.0-mm × 15-mm balloon. A 2.75-mm × 16-mm Cre8 drug-eluting stent (DES) (Alvimedica, Istanbul, Turkey) was implanted. Then, stent postdilatation with a 3.0-mm × 15-mm noncompliant balloon (18 atm) was performed and Thrombolysis in Myocardial Infarction grade 3 coronary flow was achieved (**Figures 4A and 4B**). Anatomic catheter ablation was performed in the aortic cusp and endocardial LVOT, which were the sites adjacent to the LV-summit PVC origin. The ablation catheter was advanced as close as possible to the earliest activation site under fluoroscopic and 3D anatomic mapping guidance. At the anatomically adjacent site, ablation was initiated at 30 W and the RF energy was titrated to achieve an impedance drop of at least 10 Ω to 15 Ω (typically 10%–15% drop from the baseline values). Irrigated RF applications performed in the left coronary cusp (LCC) and the endocardial LVOT site (from the aortomitral continuity) failed **(Figure 2A)**. Subsequently, an irrigated RF current was delivered in the GCV starting at 20 W, with an irrigation flow rate of 30 mL/min. Soon after the PVC was suppressed, RF delivery was continued for 60 seconds, gradually increasing the power up to 30 W to obtain an impedance drop of 10% to 15% with a maximum temperature of 42°C **(Figure 2B)**. The delivery of RF currents within the GCV and LCC was conducted under continuous fluoroscopic observation, with an angiographic catheter engaged in the left main coronary artery. The coronary artery flows were observed by manual contrast injections every 15 seconds during ablation. To avoid thermal injury, RF current was not applied within 5 mm of a coronary artery. After ablation, intravenous isoproterenol infusion (4 μg/min) was administered and burst pacing from the right ventricle, with a cycle length as short as 300 ms, was used to assess the inducibility of PVCs/VTs. No PVCs/VTs were observed during the 30-minute waiting period after RF ablation **(Figure 5)**. High-sensitivity cardiac troponin I (hs-TnI) and creatine kinase–myocardial band (CK-MB) were measured from peripheral venous blood samples; the troponin level was 0.27 ng/mL (range: 0.00–0.06 ng/mL), while the CK-MB was 16.2 ng/mL (range: 0.5–5 ng/mL) at between 12 hours and 24 hours postoperatively. There was no chest pain, PVCs, or ST-segment deviation in the ECG. The increases in Hs-TnI and CK-MB were attributed to RF ablation. Three months later, the patient was asymptomatic and the 24-hour ambulatory ECG monitoring showed a PVC burden of 0.1% without any antiarrhythmic agent.

## Discussion

In this case, we present a stepwise treatment approach to an epicardial PVC of LV summit origin with an adjacent critical coronary artery stenosis. The LV summit is the common origin of epicardial idiopathic VAs and LV-summit PVCs characteristically show either a right or left bundle branch block (RBBB and LBBB, respectively) morphologic configuration in V1 and early precordial transition between V1 and V3. Tall R-waves in the inferior leads and an S-wave in lead I, with a maximum deflection index of greater than 0.55, are also prominent features.^6^ To predict successful epicardial ablation, three ECG criteria were proposed by Santangeli et al., which include the absence of a Q-wave in V1, an R/S ratio of more than 2 in V1, and an avL/avR Q-wave ratio of 1.85. A case meeting at least two out of these three criteria suggests successful epicardial ablation has been achieved (sensitivity: 100%; specificity: 72%).^10^ An RBBB with a transition zone of V1 or less, an avL/avR Q-wave ratio of greater than 1.1, and an S-wave in V5 or V6 predicts whether an LV-summit PVC could be ablated in the accessible area. Reversal of R-wave transition in V1 (LBBB) at a more distal GCV in the inaccessible zone is possible.

Similar to these data, the clinical PVC morphology of our case on the admission ECG was consistent with an LV summit origin. Successful catheter ablation of LV-summit PVCs has been reported through the CVS from the endocardial LVOT and using a percutaneous epicardial approach.^5,11^ The aortomitral continuity, LCC, and apical LVOT are the most commonly preferred routes for the anatomic approach to catheter ablation of LV-summit PVCs. In such locations, the extent of proximity to the epicardial origin can predict the success of catheter ablation. Notably, the study by Shirai et al. reported successful outcomes of an anatomic catheter ablation performed less than 12.8 mm from the epicardial origin in a case of LV-summit PVCs.^8^

For LV-summit PVC cases experiencing unsuccessful epicardial and anatomic catheter ablations, alternative ablation strategies have been also reported, such as simultaneous unipolar and bipolar ablation from the two adjacent anatomic sites,^12,13^ half-normal saline use,^14^ or retrograde coronary venous ethanol infusion.^15^ These techniques might lead to successful ablations especially in patients with intramural–originated arrhythmias. The most serious risks of ablation from the epicardial surface are coronary events such as occlusion associated with vasospasm, dissection, and plaque rupture. Pathophysiological mechanisms such as vasospasm caused by heat dissipation, plaque rupture due to inflammatory fibrous intimal changes, and secondary thromboembolism may play a role in coronary artery damage.^16^ The case series by Steven et al. did not include permanent coronary artery stenosis in the catheter ablations performed through the GCV, but three patients with acute coronary vasospasm responded to intracoronary nitroglycerine.^17^ Our case had severe coronary artery stenosis adjacent to the LV-summit PVC origin. Despite a safety distance of greater than 5 mm between the coronary artery stenosis in the proximal LCX and the ablation point, PCI was performed first on the coronary artery stenosis in the proximal LCX, in consideration of the potential coronary vasospasm effect. Because the earliest ventricular activation was observed in the epicardial LV summit region during the PVC and because the PVC did not originate from the myocardial region fed by the LCX coronary artery, ischemic etiology was not considered in our case. Furthermore, there was no evidence of ischemia in the stress test. The PVC coupling interval (ie, the distance between the onset of the preceding sinus QRS and that of the premature beat) was calculated and varied during 24-hour ambulatory ECG monitoring. Because the PVCs exhibit variable coupling intervals, increased abnormal automaticity is more likely to be the source of the rhythm disturbance.^18,19^ Therefore, sequential PCI and RF ablation were performed in the same session.

The implantation of DESs into the vascular wall is followed by de-endothelialization, crushing of the plaque, and stretching of the vascular wall. An inflammatory process starts quickly with platelet and fibrin accumulation occurring at the injury site.^20^ To our knowledge, there are no data available in the literature regarding the effect of RF catheter ablation on this acute inflammatory process right after DES implantation. Therefore, we decided to first perform anatomic catheter ablation in the aortic cusp and endocardial LVOT as the sites adjacent to the LV-summit PVC origin. However, successful ablation could not be achieved in these areas. Thereafter, catheter ablation was successfully performed through the GCV because the patient was symptomatic under medical treatment, PVC-induced cardiomyopathy was observed and the ablation point was observed to be more than 5 mm away from the coronary artery. During ablation, we increased the irrigation flow rate up to 30 mL/min to achieve the desired level of power delivery without abrupt impedance or temperature rise. We believe that potential coronary artery damage during catheter ablation was avoided with this approach.

## Conclusion

We performed stepwise catheter ablation on the LV-summit PVC origin site adjacent to severe coronary artery stenosis using a 3D electroanatomic mapping system in a single case. Special precautions should be taken to avoid coronary artery damage during ablation from distal CVS. This is the only way to increase the success of catheter ablation and avoid potential complications.
